# Atypical Femoral Fractures in Adult Patients with Classical Osteogenesis Imperfecta

**DOI:** 10.1007/s00223-026-01576-w

**Published:** 2026-07-18

**Authors:** Mikolaj Bartosik, Oskar Windels, Florian Barvencik, Michael Amling, Ralf Oheim

**Affiliations:** Department of Osteology and Biomechanics, University Medical Center Hamburg-Eppendorf, Lottestrasse 59, 22529 Hamburg, Germany

**Keywords:** Osteogenesis imperfecta, Atypical femoral fractures, Fractures, Bisphosphonates

## Abstract

Atypical femoral fractures (AFF) are rare fractures with characteristic radiographic features, most commonly associated with long-term bisphosphonate use. Their occurrence in patients with osteogenesis imperfecta (OI), a hereditary bone disease leading to bone fragility, is not well understood. In this study, 138 adults with genetically confirmed classical OI were screened for AFF. Five patients with AFF were identified and compared to an age- and treatment-matched adult OI cohort without AFF (*n* = 23). Demographical parameters, biochemical markers, bone mineral density (DXA), bone microarchitecture (HR-pQCT), and radiographs were analyzed. In addition, antiresorptive therapy and the duration of treatment were determined and compared. In the screened OI cohort, AFF prevalence was 3.6%. No significant differences were observed between groups regarding age, weight, height, BMI, fracture history, bone mineral density, or antiresorptive therapy exposure and duration. HR-pQCT showed no significant microarchitectural differences, although a trend toward higher cortical thickness was noted in AFF patients. AFF are a rare complication in adults with classical OI and appear to be multifactorial in origin. Our findings suggest that AFF are not exclusively related to antiresorptive therapy but may be influenced by disease-specific factors, particularly the underlying collagen defect, femoral deformities and altered biomechanics. Individualized management strategies are essential, and further studies are needed to clarify underlying mechanisms and best treatment options.

## Introduction

Atypical femoral fractures (AFF) represent a rare form of pathological fractures with distinct clinical presentation. They are defined by the American Society for Bone and Mineral Research (ASBMR) and are described as low-energy femoral fractures that show characteristic radiological hallmarks, such as localized lateral cortical thickening and a medial spike [1]. While AFF have been most described in postmenopausal women receiving long-term bisphosphonate treatment for osteoporosis, their occurrence in patients with osteogenesis imperfecta (OI) is scarce and the literature often limited to case reports [2–6] or case series [7, 8].

OI is a genetically heterogeneous disorder caused by pathogenic variants in genes involved in collagen synthesis, processing, and bone mineralization [9, 10]. To date, more than 20 OI types have been described, reflecting the broad molecular heterogeneity of the disease [9]. The classical OI types I to IV originate from the Sillence classification [11], which was established based on clinical and phenotypic characteristics rather than molecular mechanisms. Classical OI, the most common inherited bone disorder, is caused by pathogenic variants in the *COL1A1* or *COL1A2* genes, which encode the α-chains of type I collagen, an important structural component of the bone matrix. This leads to an increased bone fragility with the OI subtype depending on the phenotype [10, 11]. Several factors may contribute to AFF in OI beyond pharmacologic therapy. Skeletal deformities such as femoral bowing, altered torsion, and structure deterioration change the distribution of mechanical stresses along the femoral shaft, potentially creating sites susceptible to stress accumulation and atypical fractures [8]. This biomechanical vulnerability may be exacerbated by chronic microdamage, impaired bone remodeling, or abnormal bone material properties inherent to OI. Risk factors for AFF include prolonged bisphosphonate use, which is frequently employed as the mainstay pharmacological therapy in moderate to severe OI. This raises important diagnostic and therapeutic considerations.

In this study, we present a cohort of adult classical OI patients with AFF. By examining fracture morphology and treatment history, with particular attention to long-term bisphosphonate treatment, we aim to better understand the interplay between OI and AFF. This may contribute to a more nuanced perspective on AFF for this vulnerable patient population.

## Methods

### Study Design

**Fig. 1:**
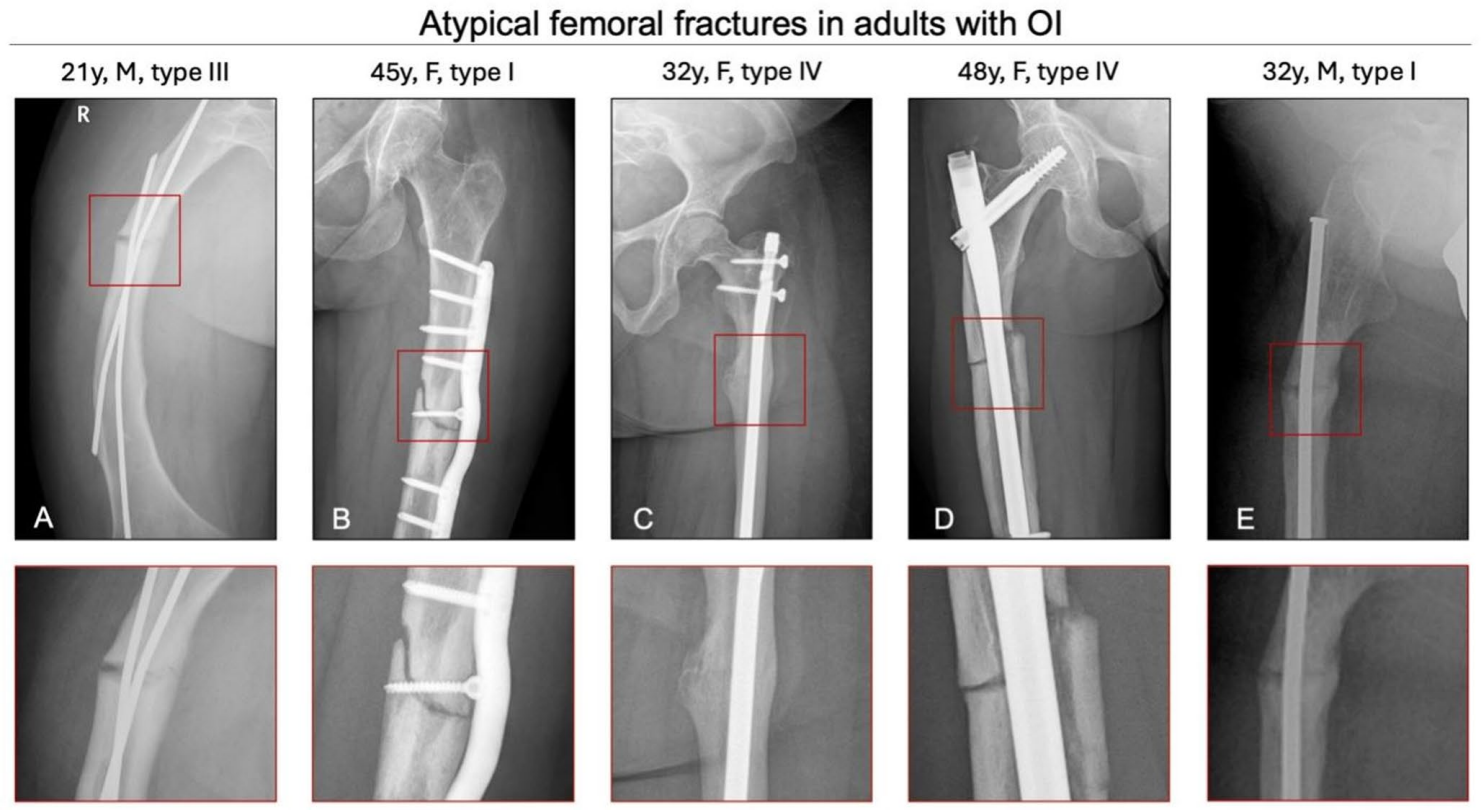
X-ray images of individual adult patients with classical OI who had an atypical femoral fracture (AFF). Above each individual X-ray, the corresponding age in years, sex (F: female; M: male), and OI type are indicated

This retrospective study was conducted in accordance with the local ethics committee (PV5364 and 2023-101124-BO-ff) and the Declaration of Helsinki. A total of 138 adult OI patients (≥ 18 years) who presented to our osteology specialty outpatient clinic (National Bone Board) were screened for a history of atypical femoral fracture (AFF) based on medical records and clinical documentation. Radiographic imaging of femoral fractures was available in 24 adult patients with OI. Overall, 5 patients were confirmed to have an AFF according to ASBMR criteria (Fig. 1). We compared the classical OI with AFF cohort with an age-and treatment matched classical adult OI cohort (*n* = 23) without a medical history of AFF. All patients were clinically diagnosed with classical OI confirmed by the detection of (likely) pathogenic variants in *COL1A1* or *COL1A2.* The patients underwent biochemical analyses, bone density measurements using dual-energy X-ray absorptiometry and bone microarchitecture measurements using high-resolution peripheral quantitative computed tomography. Furthermore, the X-rays of atypical femoral fractures of all patients were analyzed. The age at onset of the first fracture and the number of vertebral and peripheral fractures were determined using a medical history questionnaire.

## Biochemical Analysis

Blood and urine samples were routinely analyzed at the local laboratory for the following parameters: serum calcium, phosphate, alkaline phosphatase (ALP), creatinine, bone-specific alkaline phosphatase (b-ALP), osteocalcin, parathyroid hormone (PTH), 25-hydroxyvitamin D (25(OH)D), and urinary deoxypyridinoline per creatinine (DPD/Crea).

## Dual-energy X-ray Absorptiometry (DXA)

The areal bone mineral density (aBMD) was measured using dual-energy X-ray absorptiometry (Lunar iDXA, GE Healthcare, Madison, Wisconsin, USA). Measurements of aBMD were performed at both proximal femora (femoral neck and total hip) as well as at the lumbar spine (L1–4). The lowest T-score, along with the corresponding aBMD and Z-score, were then determined and used for further statistical analysis. Daily calibration scans were performed using a phantom in accordance with the manufacturer’s recommendations. Accuracy testing included calculations of the smallest significant difference based on the guidelines of the International Society for Clinical Densitometry (ISCD).

## High Resolution Peripheral Quantitative Computed Tomography (HR-pQCT)

To investigate three-dimensional bone structure parameters, patients were examined using either first-generation (XCT1) or second-generation (XCT2) HR-pQCT scans (XtremeCT and XtremeCT II, Scanco Medical AG, Bruettisellen, Switzerland) of the non-dominant distal radius and the contralateral distal tibia. Each HR-pQCT scan was performed using the manufacturer’s protocol for the standard in vivo scan (XCT1: 59.4 kVp, 900 µA, 100 ms integration time, 82.0 μm voxel size; XCT2: 68.0 kVp, 1,470 µA, 43 ms integration time, 60.7 μm voxel size). The scan range comprises 110 slices for XCT1 and 168 slices for XCT2. This results in a total scan range of 9.0 mm and 10.2 mm, respectively. In one patient in the AFF group and three patients in the control group, it was not possible to perform such a measurement in the tibia (and in some control cases also in the radius), due to metal implants or bone deformities. The HR-pQCT parameters were further standardized for analysis using device-, age-, and gender-specific reference values [12, 13] to enable comparisons.

### Statistical Analysis

For statistical analysis we used JASP 0.19.1 (University of Amsterdam, Netherlands) and GraphPad Prism 10.4.1 (GraphPad Software, San Diego, California, USA). The results are presented as the mean ± standard deviation (SD). For the HR-pQCT parameters, the percentage of the median (%Median) of the reference values was reported. To evaluate the normal distribution of the data, the Shapiro-Wilk test was used. Comparisons between two groups were performed using an unpaired, two-tailed t-test for normally distributed data and the Mann–Whitney U test for non-normally distributed data. Differences in the distribution between subgroups were tested using the chi-square test or Fisher’s exact test.

## Results

### Characterization

The characteristics of the overall cohort are summarized in Table 1. No significant differences were observed between groups in age, weight, height, BMI, lowest T-score, or fracture rates. OI types I, III and IV were evenly distributed (*p* = 0.102). Among patients with OI and an AFF, 2 out of 5 (40%) received antiresorptive therapy, compared to 7 out of 23 (30%) in the control OI cohort without AFF (*p* > 0.999). The duration of therapy did not differ significantly between groups. Detailed characterization of OI type and genetic variant in the AFF cohort is provided in Table 2. The mean time interval from atypical fracture to skeletal assessment was 3.1 ± 4.5 years. Radiographic analysis revealed femoral varus deformity in all AFF cases. The classical pattern of subtrochanteric diaphyseal fracture with medial and lateral cortical reaction was observed in all patients (Fig. 1A–E). Femoral imaging for the contralateral site was available for all five OI patients with AFF. Four of those OI patients showed cortical sclerosis of the contralateral femoral shaft consistent with a stress reaction, whereas one OI patient developed a contralateral AFF during follow-up, corresponding to a bilateral AFF rate of 20%.

**Table 1:** Overview of the total cohort of adult classical OI patients categorized the occurrence of atypical femoral fractures (AFF)

Parameter	AFF (*n* = 5)				Control (*n* = 23)				*p*-value
	Mean	SD	Min	Max	Mean	SD	Min	Max	
Demographics									
Women (n / %)	3/60%				11/48%				> 0.999
Age (years)	35.6	11.0	21.0	48.0	34.4	11.4	18.0	55.0	0.838
Weight (kg)	57.3	20.2	43.0	93.0	70.8	22.5	30.0	127	0.086
Height (m)	1.38	0.18	1.21	1.60	1.58	0.19	1.20	1.84	0.069
BMI (kg/m^2^)	29.9	7.3	21.5	36.6	28.3	8.0	19.0	47.2	0.694
DXA									
Lowest T-score	− 3.3	1.1	− 4.2	− 1.6	− 3.5	1.0	− 5.4	− 1.4	0.683
Lowest Z-score	− 2.8	1.2	− 3.9	− 1.0	− 3.2	0.9	− 4.9	− 1.2	0.509
Lowest aBMD	0.759	0.183	0.509	0.998	0.809	0.107	0.558	0.999	0.586
Fractures									
Total fractures	18	12	4	37	15	7	6	30	0.526
Vertebral fractures	10	9	0	20	4	4	1	15	0.371
Peripheral fractures	8	9	1	19	9	5	1	25	0.599
Antiresorptive treatment									
Distribution (n / %)	2/40%				7/30%				> 0.999
Duration (years)	3	4	0	9	2	4	0	12	0.566

SD, standard deviation; BMI, Body mass index; DXA, dual-energy X-ray absorptiometry; aBMD, areal bone mineral density

**Table 2:** Further characterization of adult classical OI with atypical femoral fractures (AFF)

Patient	Sex	Age at fracture	OI type	Variant	Genetical information	Antiresorptive treatment?	Treatment duration (y)
1	F	48	4	*COL1A2*	c.838G > A; p.(Gly280Ser)	yes	8
2	F	45	1	*COL1A2*	c.1009G > A; p.(Gly337Ser)	no	–
3	M	21	3	*COL1A1*	c.2101G > A; p.(Gly701Ser)	yes	7
4	M	32	1	*COL1A2*	c.858 + 1G > A; p.?	no	–
5	F	32	4	*COL1A2*	c.1783G > A; p.(Gly595Ser)	no	–

Patients were classified by sex, age at fracture, OI type, affected gene, DNA and protein change, antiresorptive treatment and treatment duration. F, female; M, male

**Table 3:** Comparison of laboratory parameters

Laboratory parameters		AFF		Control		
	Reference ranges	Mean	SD	Mean	SD	*p*-value
Calcium (mmol/l)	2.18–2.60	2.36	0.08	2.37	0.14	0.802
Phosphate (mmol/l)	0.77–1.65	1.10	0.15	0.95	0.16	0.092
ALP (U/l)	46–116	95.6	38.7	91.0	20.1	0.812
Osteocalcin (µg/l)	5.4–59.1	18.3	3.6	24.2	11.3	0.215
25(OH)D (µg/l)	> 30	24.5	11.8	27.1	17.6	0.954
b-ALP (µg/l)	5.2–24.4	19.2	12.6	19.5	9.5	0.961
PTH (ng/l)	17.4–80.1	46.1	14.8	52.7	28.2	0.520
DPD/Crea (nmol/mmol)	3–7	8	2	8	2	0.739

ALP, alkaline phosphatase; 25(OH)D: 25-hydroxyvitamin D b-ALP, bone specific alkaline phosphatase; PTH, parathyroid hormone; DPD/Crea, deoxypyridinoline per creatinine in the urine. There were no significant differences

## Bone Turnover Markers

Overall, no significant differences were found between adult patients with classical OI who had AFF and those who did not. Regarding laboratory parameters, both groups exhibited vitamin D insufficiency, although no significant difference was observed between the AFF and control cohorts (24.5  ± 11.8 µg/l in AFF vs. 27.1 ± 17.6 µg/l in control, *p* = 0.954). Additionally, elevated bone resorption, as measured by DPD/creatinine, was noted in both cohorts (8 ± 2 nmol/mmol in AFF vs. 8 ± 2 nmol/mmol in control, *p* = 0.739). However, these measurements were not obtained at the time of fracture in all patients, which limits conclusions regarding bone turnover status at the time of AFF.

### Bone Mineral Density and Bone Microarchitecture

**Fig. 2:**
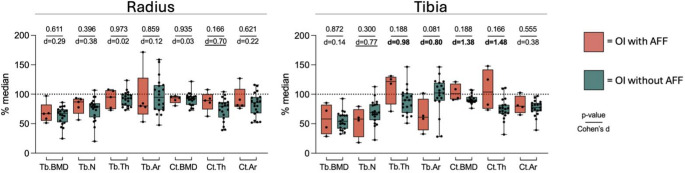
Comparison of bone microarchitecture in adult classical OI patients with and without atypical femoral fractures (AFF). Reduced trabecular and cortical bone microarchitecture was detected in both distal radius and tibia in patients with (red) and without AFF (green). HR-pQCT values for the distal radius and tibia were compared with the median device-, age-, and gender-specific reference values and expressed as a percentage of the median (XCT1: Burt et al. [12], XCT2: Whittier et al. [13]). Tb.BMD: trabecular bone mineral density; Tb.N: trabecular number; Tb.Th: trabecular thickness; Tb.Ar: trabecular area; Ct.BMD: cortical bone mineral density; Ct.Th: cortical thickness; Ct.Ar: cortical area. There were no significant differences. Exact *p*-values are shown above the comparison line. Effect sizes are reported as Cohen’s d below the comparison line, with medium effect sizes underlined and large effect sizes shown in bold

We investigated bone microarchitecture in adult OI patients with and without AFF to identify potential differences that might represent risk factors for AFF. We observed no significant differences in bone structural parameters between both groups at either the distal radius or distal tibia (Fig. 2). Both groups exhibited reduced trabecular and cortical bone microarchitecture, accompanied by decreased trabecular BMD. Notably, adults with OI and AFF tended to show higher cortical thickness and cortical area at the radius and tibia, although these differences were not statistically significant. However, moderate to strong effect sizes were observed, particularly for cortical thickness (Ct.Th) at the distal radius and tibia, suggesting that these differences may still be relevant.

## Discussion

AFF in adult OI patients are rarely described but represent an important clinical challenge in clinical management. In our cohort of 138 adults with classical OI, we identified 5 (3.6%) patients who fulfilled ASBMR criteria for AFF [1]. Previous reports have described the occurrence of atypical femoral fractures in adult OI patients, with prevalences of up to approximately 7% [7], supporting the concept that AFF represents a rare but clinically relevant complication in this population. This prevalence is considerably higher than the incidence of AFF reported during long-term bisphosphonate therapy for postmenopausal osteoporosis [14]. Although a direct comparison between a cross-sectional prevalence and an exposure-based incidence is limited, this difference suggests that disease-specific factors beyond antiresorptive exposure may contribute to AFF susceptibility in OI.

While bisphosphonates are widely used in OI to improve bone mineral density, reduce fracture incidence, and alleviate pain [15], their long-term suppression of bone turnover has raised concerns about microdamage accumulation and atypical fracture risk [14, 16]. However, Trejo et al. reported that atypical fracture features in children and young adults with OI were not significantly associated with bisphosphonate exposure once phenotype severity was considered [8]. Moreover, in adults with OI, it was found that two out of four patients with AFF had received bisphosphonate therapy and thus antiresorptive therapy cannot be considered the primary contributing factor [7]. Rather, the severity of the underlying condition must be taken into account, as genetic stratification in osteogenesis imperfecta contributes to phenotypic variability and may influence skeletal fragility and fracture risk [17, 18]. Indeed, OI-causing variants in *COL1A1/1A2*, together with variants in other genes underlying different OI types, are among the most frequently reported monogenic causes of AFF [19]. This further supports the view that a disease-related predisposition contributes to AFF in OI, independent of antiresorptive exposure. In our cohort of adult OI patients, neither the prevalence nor the duration of antiresorptive therapy differed between patients with and without AFF, suggesting that pharmacological treatment alone is unlikely to fully explain the occurrence of AFF in adults with OI. Thus, the occurrence of these atypical fractures in OI patients cannot be attributed solely to bisphosphonates and should always be considered in combination with the severity of the disease, bone deformities, and treatment. These findings therefore support the concept that additional disease-related or mechanical factors may contribute to AFF susceptibility in this population.

Mechanical factors such as bone deformities, especially femoral bowing, should also be taken into consideration. Bone deformities represent a central risk factor even for patients without hereditary bone disorders [20–22] and may substantially alter biomechanical loading along the femoral shaft, although bone geometry alone has not consistently proven to be a strong predictor of AFF in bisphosphonate-associated cases [23]. In OI, however, femoral deformities are typically far more pronounced. Moreover, the structurally abnormal type I collagen and the resulting impaired bone material properties may further contribute to AFF susceptibility, independent of geometry alone. Notably, all OI patients with AFF exhibited relevant femoral bowing, supporting the hypothesis that bone deformities may predispose to atypical fracture patterns. Given the predisposition to AFF in cases of deformed femora, surgical correction may be considered [7]. Corrective osteotomies and rodding procedures have long been integral in OI management to prevent recurrent fractures, restore alignment, and consequently preserve mobility [24, 25]. Nevertheless, AFF incidence in OI highlights the importance of individualized treatment strategies, balancing the benefits of antiresorptive therapy while also addressing mechanical deformities where appropriate. HR-pQCT analyses at the distal radius and tibia did not reveal statistically significant differences between groups. However, patients with AFF tended to exhibit lower trabecular number together with higher trabecular thickness and increased cortical density and thickness. Although these findings must be interpreted cautiously due to the small sample size, this pattern may reflect altered bone remodeling and structural adaptation, which could influence mechanical stress distribution and susceptibility to atypical fracture patterns in OI.

**Fig. 3:**
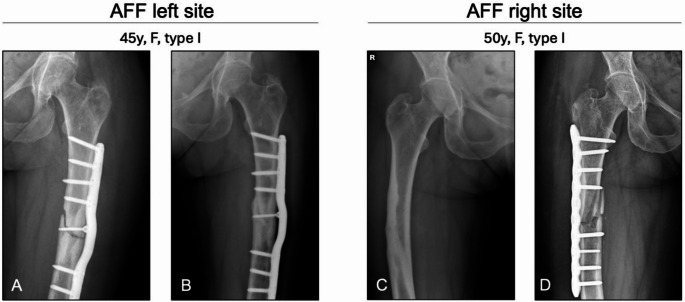
Patient report of an adult female with OI type I and AFF. The patient presented with a nonunion of AFF (**A**). One year after the first consultation and initial surgical treatment with plate osteosynthesis elsewhere, a fracture nonunion was diagnosed, and we initiated an anabolic therapy with teriparatide, which led to fracture healing and clinical improvement with diminishing pain (**B**). However, due to high resorption rates after teriparatide treatment, one year of antiresorptive therapy was initiated. The patient subsequently suffered an AFF on the opposite side five years later (**C** and** D**)

From a therapeutic view, AFF in OI require a multimodal approach. In addition to surgical treatment and prevention mentioned above, it has been reported that anabolic therapy with teriparatide promotes fracture healing and improves bone turnover in OI-related AFF [6, 26]. In one patient with OI type I (treatment-naïve) and AFF from our cohort, osteoanabolic therapy was initiated following AFF with non-union. Initial surgical treatment consisted of plate osteosynthesis, as intramedullary nailing was not feasible due to bone deformity and severe bone sclerosis (Fig. 3A). One year later, the patient presented with persistent pain in the area of the AFF, where a delayed fracture union was observed, at which point osteoanabolic therapy with teriparatide was initiated. This was associated with favorable fracture healing and reduction in pain (Fig. 3B). Nevertheless, after teriparatide therapy and subsequent short term antiresorptive treatment due to high bone resorption, she suffered an AFF on the opposite side (Fig. 3C and D), highlighting the importance of multiple factors in the development of AFF and the need for individualized treatment strategies. In our OI cohort, all five AFF patients received an X-ray scan of the contralateral femur or a pelvic overview radiograph showing extensive portions of the proximal femurs, in accordance with ASBMR recommendations [1]. In this context, it has been shown that also an extended DXA examination of the femur can detect cortical bulging as a prodromal sign of incomplete AFF and may serve as a practical screening tool [27]. All five patients of our cohort were asymptomatic and did not show incomplete AFF but increased cortical thickness and sclerosis of the contralateral femoral shaft consistent with a chronic stress reaction related to femoral curvature and the underlying bone disease. Only one patient developed contralateral AFF in the further course without first expressing local complaints (Fig. 3). This corresponds to a contralateral AFF rate of 20% (1/5) in our OI cohort. These findings suggest that contralateral imaging should be considered in all OI patients with AFF in accordance with the ASBMR recommendations. Whether prophylactic surgical stabilization is warranted must be determined on an individual basis. In OI, the complex femoral anatomy, pre-existing deformities, and high revision rates associated with intramedullary fixation require careful patient selection and interdisciplinary planning [25]. Although the evidence base for OI has so far been limited to case reports, this therapeutic approach could be of particular interest given the context of impaired remodeling and altered bone mechanics. However, larger studies are required to better define its role in OI-associated AFF.

This study has several limitations that should be acknowledged. First, the sample size was relatively small, which partly reflects the rarity of OI and the limited availability of well-characterized patient cohorts. Second, femoral deformities in the control group could not be systematically assessed, which may limit direct comparability between groups with regard to femoral morphology. Third, the time point of the examinations varied in relation to the onset of AFF, since some patients were examined months or years after the fracture occurred. This delay may have influenced the biochemical, densitometric, and microarchitectural findings and limits the interpretation of factors potentially related to the development of AFF at the time of fracture. Only Caucasian patients were included in this study, which limits the generalizability of the findings and does not allow conclusions to be drawn for other racial or ethnic groups. Despite these limitations, the study has notable strengths. In particular, we performed a comprehensive skeletal assessment combining DXA-derived bone mineral density measurements, detailed evaluation of bone microstructure, and analysis of bone turnover markers. This integrative approach allowed for a multidimensional characterization of skeletal properties beyond conventional bone density measurements.

## Conclusion

In conclusion, AFF in patients with OI appear to be rare events. Although antiresorptive therapy was present in approximately 40% of cases, these fractures cannot be attributed solely to bisphosphonate exposure. Rather, the occurrence of AFF in this population likely reflects a complex interplay of factors, including disease severity, the structurally abnormal type I collagen with consequently impaired bone material properties and underlying bone deformities. Future studies with larger cohorts are needed to better characterize the incidence, risk factors, and potential predisposing conditions for atypical femoral fractures in patients with osteogenesis imperfecta, in order to improve risk stratification and clinical management.

## Data Availability

The relevant data is published in the manuscript. However, further data can be requested from the corresponding author upon reasonable request, but due to data protection regulations, restrictions may apply.
